# Genome-Wide Expression Analysis in Fibroblast Cell Lines from Probands with Pallister Killian Syndrome

**DOI:** 10.1371/journal.pone.0108853

**Published:** 2014-10-16

**Authors:** Maninder Kaur, Kosuke Izumi, Alisha B. Wilkens, Kathryn C. Chatfield, Nancy B. Spinner, Laura K. Conlin, Zhe Zhang, Ian D. Krantz

**Affiliations:** 1 The Division of Human Genetics, The Children's Hospital of Philadelphia, Philadelphia, Pennsylvania, United States of America; 2 Research Center for Epigenetic Disease, Institute of Molecular and Cellular Biosciences, The University of Tokyo, Tokyo, Japan; 3 Department of Pediatrics, Section of Pediatric Cardiology, The Children's Hospital of Colorado, Denver, Colorado, United States of America; 4 The Department of Pathology, The Children's Hospital of Philadelphia, Philadelphia, Pennsylvania, United States of America; 5 The Perelman School of Medicine at The University of Pennsylvania, Philadelphia, Pennsylvania, United States of America; 6 Center for Biomedical Informatics, The Children's Hospital of Philadelphia, Philadelphia, Pennsylvania, United States of America; Queen's University Belfast, United Kingdom

## Abstract

Pallister Killian syndrome (OMIM: # 601803) is a rare multisystem disorder typically caused by tissue limited mosaic tetrasomy of chromosome 12p (isochromosome 12p). The clinical manifestations of Pallister Killian syndrome are variable with the most common findings including craniofacial dysmorphia, hypotonia, cognitive impairment, hearing loss, skin pigmentary differences and epilepsy. Isochromosome 12p is identified primarily in skin fibroblast cultures and in chorionic villus and amniotic fluid cell samples and may be identified in blood lymphocytes during the neonatal and early childhood period. We performed genomic expression profiling correlated with interphase fluorescent in situ hybridization and single nucleotide polymorphism array quantification of degree of mosaicism in fibroblasts from 17 Caucasian probands with Pallister Killian syndrome and 9 healthy age, gender and ethnicity matched controls. We identified a characteristic profile of 354 (180 up- and 174 down-regulated) differentially expressed genes in Pallister Killian syndrome probands and supportive evidence for a Pallister Killian syndrome critical region on 12p13.31. The differentially expressed genes were enriched for developmentally important genes such as homeobox genes. Among the differentially expressed genes, we identified several genes whose misexpression may be associated with the clinical phenotype of Pallister Killian syndrome such as downregulation of *ZFPM2*, *GATA6* and *SOX9*, and overexpression of *IGFBP2*.

## Introduction

**Figure 1:**
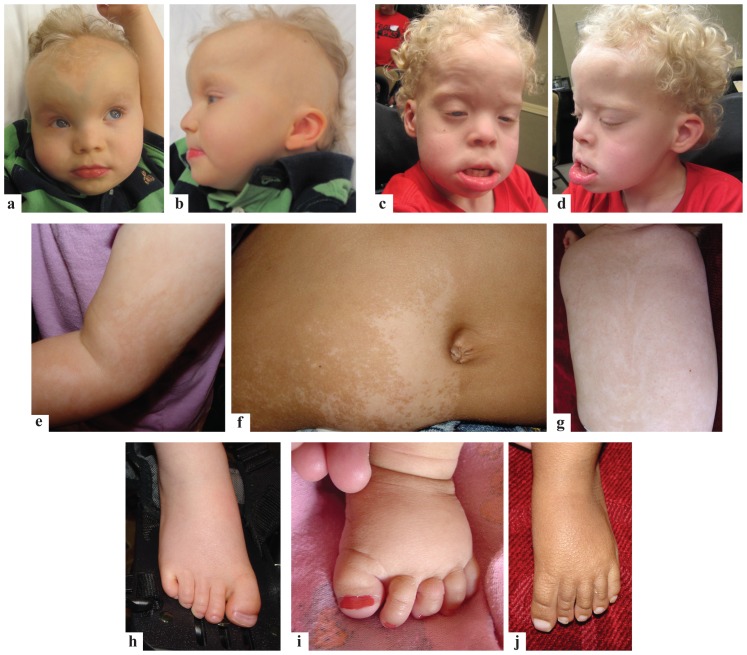
Clinical features of PKS. (A)–(D). Full face and profile views of the facial features in two unrelated children with PKS. (E)–(G). Swirly hypo- and hyperpigmentation of the skin in 3 children with PKS. (H)–(J). Broad first toes in 3 children with PKS.

Originally described by Pallister et al. [1], [2] and later by Killian and Teschler-Nicola, Pallister Killian Syndrome (PKS) (OMIM #601803) also known as tetrasomy 12p and isochromosome 12p mosaicism is a rare chromosomal aneuploidy with an estimated prevalence of 1/20,000 [3], [4]. PKS is characterized by tissue-specific mosaicism of a supernumerary isochromosome which is typically a metacentric chromosome composed of material from the short arm of chromosome 12: i(12)(p10) [5]. Most commonly, individuals with PKS have four copies of the complete short arm of chromosome 12, although rarely, probands may have a complete or partial duplication of 12p and still manifest the full PKS phenotype [6]. We have recently defined a PKS minimal critical region mapping to 12p13.31 [7]. There have been more than 150 reported probands (reviewed in Wilkens et al.) [8], [9]. Probands manifest a very recognizable pattern of malformations however there is a wide range of variability in phenotypic expression (Figure 1). Typical features include cognitive impairment, prenatal overgrowth followed by postnatal growth deceleration, hypotonia, seizures, cutaneous hypo and/or hyperpigmentation, facial dysmorphia including a high forehead, broad nasal bridge, telecanthus, sparse fronto-temporal hair at birth, high arched or cleft palate, posterior helical ear pits, short neck, supernumerary nipples, limb and genitourinary anomalies, congenital heart defects and diaphragmatic hernias [9].

Isochromosome 12p is seen mainly in skin fibroblast cultures and in chorionic villus and amniotic fluid cell samples but can be identified in blood lymphocytes during the neonatal and early childhood period [10], [11]. The isochromosome is often lost in the peripheral blood, presumably due to a selective growth advantage of the karyotypically normal disomic cell populations in the bone marrow of PKS individuals [12]. The percentage of tetrasomic cells does not correlate with severity of the syndrome, the patient's longevity, or degree of mental retardation [9], [13], [14]. The prevalence of cells with an extra metacentric chromosome i(12)(p10) is usually highly variable and tissue-limited or tissue-specific mosaicism is a well-described characteristic of PKS. Diagnosis of PKS, especially in those individuals beyond the neonatal period, traditionally requires a skin biopsy and analysis of fibroblasts.

The constellation of structural and neurocognitive deficits seen in PKS in a highly conserved manner, coupled with a narrow minimal critical region delineated on 12p13.31 would suggest that a few genes (or even a single gene) within this region that are critical regulators of human development are responsible for the PKS phenotype when present in extra copies. In order to effect the pleiotropic manifestations observed in PKS the critical gene(s) on 12p are likely to be key regulators of downstream genes or gene networks important for the growth and differentiation of the tissues affected in this diagnosis. In order to begin to delineate the downstream 12p effector genes in PKS we used the Affymetrix Human Genome U133 plus 2.0 arrays to perform a genome-wide expression analysis in 17 probands with PKS and 9 age, gender and race matched controls. This analysis was able to identify 354 genes that were statistically differentially expressed in PKS probands, 180 of which were up- and 174 down-regulated with at least a 1.5 fold change and a p value less than 0.05. The most statistically significantly dysregulated genes on 12p mapped to 12p13.31, a region previously described cytogenetically to represent a PKS minimal critical region [7]. The non-12p genes that were most significantly dysregulated include a large number of homeobox genes and other transcription factors critical in mammalian development.

## Materials and Methods

### Study population

Fibroblast cell lines from a cohort of 17 probands (5 females and 12 males) with PKS and 9 age, gender and race matched normal controls (5 females and 4 males) were used (Table 1). The control fibroblast cell lines were obtained from the Coriell Cell Repository (http://ccr.coriell.org/). All patients and families enrolled in this study were done so in writing by following a specific approval for this study from the Institutional Review Board at The Children's Hospital of Philadelphia by the Committee for the Protection of Human Subjects (approval # IRB 10-007476, DHHS Federal Wide Assurance Identifier: FWA0000459). For minors and those individuals unable to consent themselves due to intellectual disability their guardians or appointed caretakers provided consent. Consents were obtained in writing for the publication of photographs of individuals depicted in the figure. One or more experienced clinical dysmorphologists evaluated all the affected individuals. Sample processing was performed in two batches, batch I comprised 8 patients and 5 controls and batch II had 9 patients and 4 controls.

**Table 1:** Sample/mosaic ratio information.

Sample ID	Passage	Split Ratio	Population db time	Gender	Age	Ethnicity	Mosaicism % in Fibroblast(short term culture)	Mosaicism % in Fibroblasts (long term)	Mosaicism % in Blood	Mosaicism % By FISH	Mechanism of origin
MAC105P	9	1∶4	18	F	1month	Caucasian	55%	NA	65%	NA	NA
MAC192P	9	1∶4	18	F	4 yrs	Caucasian	60%	45%	5%	43%	MII
MAC193P	8	1∶4	16	F	3 yrs	Caucasian	45%	5%	0%	4%	MII
MAC195P	10	1∶4	20	M	3 yrs	Caucasian	65%	75%	20%	75%	MII
MAC196P	8	1∶4	16	M	4 yrs	Caucasian	50%	20%	0%	20%	MII
MAC197P	12	1∶4	24	M	7 yrs	Caucasian	65%	NA	0%	50%	MII
MAC198P	9	1∶4	18	M	6 Yrs	Hispanic	50%	5%	10%	8%	MII
MAC199P	9	1∶4	18	M	1 yrs	Caucasian	30%	15%	35%	20%	MII
MAC200P	9	1∶4	18	M	5 yrs	Caucasian	100%	100%	0%	100%	MII
MAC201P	12	1∶4	24	M	5 yrs	Caucasian	75%	75%	NA	80%	MII
MAC203P	8	1∶4	16	M	5 yrs	Caucasian	80%	60%	NA	65%	MII
MAC223P	9	1∶4	18	M	Unknown	Unknown	55%	50%	0%	46%	MII
MAC232P	13	1∶4	26	F	3 yrs	Caucasian	NA	50%	NA	46%	NA
MAC239P	8	1∶4	16	M	5 yrs	Caucasian	NA	45%	NA	47%	NA
MAC240P	10	1∶4	20	M	27 yrs	Caucasian	NA	0%	NA	0%	NA
MAC241P	11	1∶4	22	M	18 months	Caucasian	NA	95%	NA	96%	MII
MAC242P	10	1∶4	20	F	3 yrs	Caucasian	NA	0%	NA	0%	NA
GM00969	16	1∶4	32	F	2 yrs	Caucasian	NA	NA	NA	NA	NA
GM01652	15	1∶4	30	F	11 yrs	Caucasian	NA	NA	NA	NA	NA
GM05399	9	1∶5	18	M	1 yrs	Caucasian	NA	NA	NA	NA	NA
GM02036	16	1∶4	32	F	11 yrs	Caucasian	NA	NA	NA	NA	NA
GM00942	12	1∶3	20.7	F	5 yrs	Caucasian	NA	NA	NA	NA	NA
GM05565	10	1∶5	22	M	3 yrs	Hispanic	NA	NA	NA	NA	NA
GM08398	10	1∶4	20	M	8 yrs	Caucasian	NA	NA	NA	NA	NA
GM08447	7	1∶5	15.6	F	2days	Caucasian	NA	NA	NA	NA	NA
GM00497	20	1∶3	34	M	4 yrs	Caucasian	NA	NA	NA	NA	NA

### Cell culture

Fibroblasts cell lines from 17 probands with PKS and nine normal controls were uniformly cultured in RPMI 1640 (Life Technologies, Carlsbad, CA, USA) supplemented with 20% FBS (Hyclone South Logan, UT, USA), antibiotics (100 units/ml of penicillin and 100 ug/ml of streptomycin), and 1% L-glutamine (Life Technologies, Carlsbad, CA, USA). Approximately 9.0–15.0×10^5^ exponentially growing cells were seeded in 15 ml media in 75 ml falcon flasks (BD Biosciences, San Jose, CA, USA). Cells were incubated at 37°C in a humidified atmosphere of 5% CO_2_. Cells were trypsinized (0.25% trypsin-EDTA) and split at 75–80% confluency. All 26-cell lines were cultured until they reached a population-doubling level between 15–35. Total RNA, and DNA were isolated from the same cultures for all the samples. Slides were also prepared for cytogenetic analyses from the same cultures. Standard G-banded karyotyping, single nucleotide polymorphism (SNP) array, and fluorescent *in situ* hybridization (FISH) analysis of 12p was performed on all cell lines at the start and end points of culturing to provide an accurate estimate of degree of mosaicism of these samples (Table 1).

### Cytogenetic analysis

Chromosome preparations from cultured fibroblasts were analyzed by FISH using a chromosome 12p specific DNA probe. The probe was labeled by nick translation with spectrum green. FISH was done according to standard protocol [15]. To validate the level of mosaicism in the probands 2 ml of cell culture from the flasks used for RNA and DNA isolation were cultured on flaskettes (Thermo scientific, Waltham, MA, USA). Slides were prepared for chromosome (interphase) analysis using standard cytogenetic protocols. For each proband 200 cells were analyzed.

We also used genome-wide single nucleotide polymorphism (SNP) arrays (Illumina HumanHap 550K; Illumina, San Diego, CA, USA) to detect percentage of mosaicism in all seventeen probands with PKS as described previously [12]. For this analysis DNA was isolated from the cultured fibroblasts (OD_260_/OD_280_ 1.8–2.0 and OD_260_/OD_230_>2.0, respectively). The samples were genotyped on the Illumina Bead Station (Illumina, San Diego, CA, USA). Analysis of all copy number variation calls was detected using Illumina Bead Studio software. The degree of mosaicism in all the probands were detected by assessing probe intensities measured by log R ratios, along with shifts in genotype frequencies of SNP probes measured by B allele frequencies [16].

### RNA isolation and sample preparation for hybridization

Total RNA was isolated from each sample using Qiagen RNeasy mini kit (Qiagen, Valencia, CA, USA). All RNA samples exhibited intact 28S and 18S ribosomal RNA on denaturing agarose gel electrophoresis and OD_260_/OD_280_ absorbance ratio fell within the acceptable range of 1.8–2.1. RNA yield was determined spectrophotometrically (Nanodrop ND-1000 spectrophotometer; Thermo scientific, Waltham, MA, USA). RNA extracts were DNase treated. For array analysis at least 3 ug of total RNA was transcribed into Double Stranded cDNA with oligo (dT)24 T7 primer using Invitrogen SuperScript Double-Stranded cDNA synthesis kit (Life Technologies, Grand Island, NY, USA). The cDNA was purified with Affymetrix GeneChip Sample Cleanup module (Affymetrix, Santa Clara, CA, USA) and used into an in vitro transcription reaction to produce labeled cRNA with Enzo Bioarray High Yield RNA Transcript Labeling Kit from Enzo Life Sciences (Farmingdale, NY, USA) and further fragmented to 35–200 bp oligos for hybridization. All steps for sample preparation and processing were performed according to manufacturer's instructions.

### Hybridization to Affymetrix GeneChip expression array and data collection

The newly synthesized and biotin labeled cRNA was fragmented by incubation in fragmentation buffer for 35 minutes at 94°C, 15 ug/30 ul of the fragmented samples were sent to the microarray facility at The Children's Hospital of Philadelphia for hybridization. Microarray hybridization was performed on commercially available high density Affymetrix HG-U133 plus 2.0 GeneChip arrays (Affymetrix, Santa Clara, CA, USA). Arrays were washed and stained according to standard affymetrix protocols using an Affymetrix Fluidics Workstation. Arrays were scanned using Affymetrix GeneArray scanner 3000 and the cell intensities (.CEL files) were captured by Affymetrix Genechip Operating Software (GCOS) as per the Affymetrix protocol. The HG U133 Plus 2.0 Arrays contains over 54,000 probe sets to analyze the expression level of more than 47,000 transcripts and variants, including approximately 38,500 well-characterized human genes. The sequences from which these probes were derived were selected from GeneBank, dnEST, and Refseq [17].

### Data analysis

All Affymetrix probes were re-grouped into unique Entrez gene IDs using custom library file downloaded from BRAINARRAY database (http://brainarray.mbni.med.umich.edu). The raw data in.CEL files were normalized and summarized by *r*obust *m*ultiarray *a*verage (RMA) method to generate a 26*17,726 matrix, where 17,726 is the number of unique Entrez genes and 26 is the number of samples. The normalized data were then log2-transformed.

The normalized data was subsequently analyzed by the unsupervised multivariate analysis tool, principal component analysis (PCA). Differential expression of genes between control and PKS samples was evaluated by the difference of group means (magnitude of change) and the p value of t test (significance of change).

### Gene functional annotation and clustering analysis

To identify biologically relevant groups of genes we performed functional gene annotation and cluster analysis using the Database for Annotation, Visualization and Integrated Discovery (DAVID) of up- and down-regulated genes (http://david.abcc.ncifcrf.). To investigate the biological functions involved in the discriminating genes we did Gene Ontology (GO) Analysis (http://www.geneontology.org). Using Non-stringent cutoffs (25% difference) and p values less than 0.05, pathway analysis was performed using Ingenuity Pathway Analysis (IPA) (Ingenuity Systems, Inc., http://www.ingenuity.com). IPA uses the knowledge base to identify interactions of input genes within the context of known biological pathways.

## Results

### Cytogenetic analysis

Levels of mosaicism of the PKS probands used in this study ranged from 0% to 100% of cells. In two samples the isochromosome could not be detected, two had a very low level of 4% and 8% and in the rest had mosaic levels ranging from 40% to 100% at the time of RNA extraction for the expression arrays (Table 1).

### Gene expression pattern in PKS

**Figure 2:**
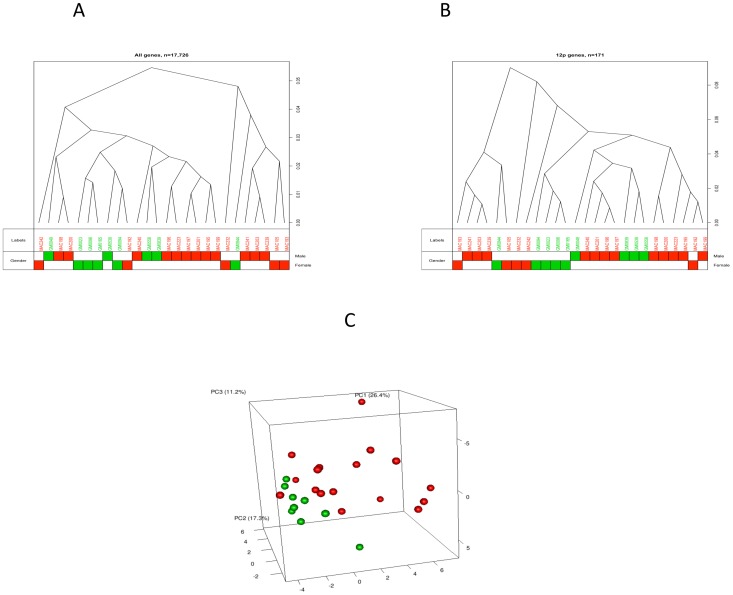
Patient and control sample clustering. Red squares/circles represent PKS patients and Green squares/circles represent control samples. (A) Unsupervised clustering of 26 samples using all genes. (B) Unsupervised clustering of 26 samples using genes located on 12p.(C) PCA result. Proportion of Variance % (PC1-24.818, p 0.001; PC2-17.814, p 0.022; PC3-0.022, p 0.772).

To test whether the probands with PKS have a unique and specific gene expression profile, we used clustering and principal component analysis. Figure 2A shows the unsupervised clustering of all 26 samples using all genes. Control and PKS samples were not distinctively separated. Gender is suggested as a confounding factor according to a large cluster of male samples clustering in the middle. Clustering of samples using expressed genes located only on 12p allowed for a more clear separation of control and PKS samples (Figure 2B). The difference among the probands likely reflects their different levels of mosaicism. Gender is an even more important factor, especially for the control samples (Figure 2B). We further explored the gene expression profile using PCA. By visualizing projections of the components identified by PCA in low dimension spaces, we were able to observe the grouping of PKS and control samples reflecting underlying patterns in their gene expression profiles (Figure 2C, Table S5). Variability among the probands likely depicts the different levels of mosaicism.

### Expression/level of mosacism

**Figure 3:**
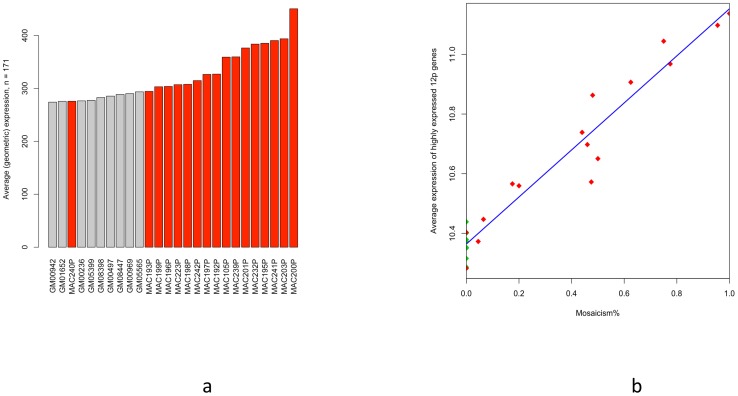
Gene expression levels in PKS. **a**: Mosaic 12p expression level correlation: Average expression of 171 genes on 12p in patients and controls. Red bars are patients and Grey is controls. X-axis represents samples and Y-axis shows average expression of genes on 12p. **b**: Level of i12p mosaicism compared to 12p gene expression: average of 64 genes with expression higher than the 3rd quartile of global expression distribution, were correlated to mosaicism%. The blue line in the figure is the fitting line of linear regression. Red diamonds are the probands and green are the controls.

To identify if any correlations existed between the expression levels of the genes on the short arm of 12 to the level of mosaicism, we compared the copy number of genes on 12p to expression level between the patients and the controls (Figure 3a). The data suggests that as long as the 12p genes are commonly expressed in healthy control samples, their expression level in patients is proportional to the level of mosaic (Figure 3b). The average expression level of 12p genes and the level of mosaicism had a Pearson's correlation of r = 0.972 (p = 5.0E-14).

### Differentially expressed genes between PKS and control groups

As a next step, we undertook the identification of genes whose expression levels are significantly different between the PKS and control groups. In general, there were a small number of genes whose expression was noticeably changed in PKS. Using non-stringent cutoffs (at 25% difference and p less than 0.05), only 354 genes (2% of genes analyzed) were differentially expressed between the two groups, 180 genes were up- and 174 genes down regulated in probands with PKS (Table S1 and S2). Up-regulated genes were highly enriched with genes located on the short arm of chromosome 12 (12p), fitting the pathology of PKS. Out of 180 up-regulated genes, 57 (32%) were located on 12p. Amongst these, genes mapping to cytogenetic band 12p13.31 were among the ones that were most significantly dysregulated in the probands compared to controls. Out of approximately 100 genes in this region, *CLEC2B*, *LOC374443* and *NCAPD2* were over-expressed by 1.5 fold in the PKS probands (Table 2). None of the down-regulated genes mapped to 12p. The magnitude of fold increase in the expression of the differentially expressed genes between the controls and the patients ranged from 0.3 to 2.4. A full list of differentially expressed genes is provided in Tables S1 and S2. These lists include many disease-associated genes, and *ZFPM2* was the most significantly down regulated gene, *GATA6* ranked 5^th^, and *SOX9* was 24^th^. *IGFBP2* was ranked second in the upregulated gene list.

**Table 2:** List of upregulated genes on 12p in PKS probands.

Symbol	Gene Name	Chr	Cytoband	StDev	Range	Mean_Ctrl	Mean_PKS	PKS-Ctrl	Fold diff	p_ttest
CLEC2B	C-type lectin domain family 2, member B	12	12p13-p12	1.211	4.942	6.051	7.194	1.142	2.207	9.63E-03
LOC374443	CLR pseudogene	12	12p13.31	0.546	1.862	6.162	6.917	0.755	1.688	6.15E-05
CCDC77	coiled-coil domain containing 77	12	12p13.33	0.593	2.279	6.790	7.441	0.651	1.571	7.67E-04
C12orf4	chromosome 12 open reading frame 4	12	12p13.3	0.448	1.802	8.439	9.084	0.645	1.564	6.77E-05
LYRM5	LYR motif containing 5	12	12p12.1	0.438	1.988	9.504	10.143	0.639	1.557	9.95E-06
PPFIBP1	PTPRF interacting protein, binding protein 1 (liprin beta 1)	12	12p11.23-p11.22	0.596	2.404	7.519	8.140	0.621	1.538	2.39E-03
NCAPD2	non-SMC condensin I complex, subunit D2	12	12p13.3	0.672	2.279	8.278	8.891	0.613	1.529	6.90E-03
MANSC1	MANSC domain containing 1	12	12p13.2	0.667	2.283	6.115	6.716	0.600	1.516	4.02E-02
RECQL	RecQ protein-like (DNA helicase Q1-like)	12	12p12	0.506	2.287	8.870	9.454	0.584	1.499	5.78E-04
CCDC91	coiled-coil domain containing 91	12	12p11.22	0.429	2.005	8.767	9.330	0.563	1.477	1.61E-03
CLEC12A	C-type lectin domain family 12, member A	12	12p13.2	0.758	3.102	4.747	5.293	0.546	1.460	2.48E-02
C12orf32	chromosome 12 open reading frame 32	12	12p13.33	0.445	1.751	8.910	9.421	0.511	1.425	9.49E-04
TAPBPL	TAP binding protein-like	12	12p13.31	0.415	1.926	7.875	8.376	0.502	1.416	8.70E-03
FGFR1OP2	FGFR1 oncogene partner 2	12	12p11.23	0.425	1.845	8.039	8.524	0.485	1.399	9.30E-04
MRPS35	mitochondrial ribosomal protein S35	12	12p11	0.360	1.450	10.438	10.920	0.482	1.396	7.37E-05
NDUFA9	NADH dehydrogenase (ubiquinone) 1 alpha subcomplex, 9, 39kDa	12	12p13.3	0.410	1.332	10.626	11.099	0.473	1.388	2.86E-04
MED21	mediator complex subunit 21	12	12p11.23	0.381	1.558	9.368	9.832	0.464	1.380	2.62E-04
DERA	2-deoxyribose-5-phosphate aldolase homolog (C. elegans)	12	12p12.3	0.375	1.315	10.019	10.480	0.461	1.377	1.14E-04
CMAS	cytidine monophosphate N-acetylneuraminic acid synthetase	12	12p12.1	0.404	1.804	8.350	8.809	0.459	1.375	1.68E-03
COPS7A	COP9 constitutive photomorphogenic homolog subunit 7A (Arabidopsis)	12	12p13.31	0.376	1.126	10.861	11.317	0.457	1.372	1.75E-04
ATN1	atrophin 1	12	12p13.31	0.442	1.762	8.908	9.364	0.456	1.372	3.44E-03
DNM1L	dynamin 1-like	12	12p11.21	0.442	1.457	9.450	9.900	0.450	1.366	3.04E-03
YARS2	tyrosyl-tRNA synthetase 2, mitochondrial	12	12p11.21	0.343	1.403	9.381	9.817	0.436	1.353	8.76E-05
PARP11	poly (ADP-ribose) polymerase family, member 11	12	12p13.3	0.284	1.095	6.035	6.462	0.427	1.345	1.31E-05
C12orf11	chromosome 12 open reading frame 11	12	12p11.23	0.453	1.554	9.662	10.083	0.421	1.339	1.78E-02
KLHDC5	kelch domain containing 5	12	12p11.22	0.374	1.664	8.630	9.041	0.412	1.330	5.14E-03
CSDA	cold shock domain protein A	12	12p13.1	0.304	1.090	12.358	12.765	0.407	1.326	4.39E-05
NECAP1	NECAP endocytosis associated 1	12	12p13.31	0.345	1.404	9.902	10.294	0.393	1.313	5.78E-04
FOXJ2	forkhead box J2	12	12p13.31	0.318	1.192	9.854	10.244	0.390	1.310	1.46E-04
ITFG2	integrin alpha FG-GAP repeat containing 2	12	12p13.33	0.275	1.179	8.798	9.185	0.387	1.308	5.67E-05
AMN1	antagonist of mitotic exit network 1 homolog (S. cerevisiae)	12	12p11.21	0.512	2.518	6.889	7.272	0.383	1.304	4.78E-02
ETNK1	ethanolamine kinase 1	12	12p12.1	0.375	1.572	6.752	7.128	0.376	1.298	5.00E-03
JARID1A	jumonji, AT rich interactive domain 1A	12	12p11	0.342	1.412	7.785	8.155	0.370	1.292	1.76E-03
KIAA0528	KIAA0528	12	12p12.1	0.392	1.609	8.924	9.292	0.368	1.291	4.70E-02
RASSF8	Ras association (RalGDS/AF-6) domain family (N-terminal) member 8	12	12p12.3	0.309	1.515	6.623	6.974	0.351	1.275	1.65E-03
DDX47	DEAD (Asp-Glu-Ala-Asp) box polypeptide 47	12	12p13.1	0.359	1.474	11.248	11.599	0.350	1.275	8.74E-03
WBP11	WW domain binding protein 11	12	12p12.3	0.398	1.401	9.404	9.753	0.350	1.274	8.61E-03
RIMKLB	ribosomal modification protein rimK-like family member B	12	12p13.31	0.415	1.882	7.446	7.794	0.348	1.273	4.40E-02
ING4	inhibitor of growth family, member 4	12	12p13.31	0.325	1.511	9.488	9.818	0.330	1.257	4.24E-03

### Validation of expression array data by ddPCR

Using the QX100 Droplet Digital PCR system (Bio-Rad, Pleasanton, CA) expression levels of 10 significantly dysregulated genes, 5 up- and 5 down-regulated genes (Table S6) were validated. RNA expression levels from all samples were measured with commercially available FAM labeled TaqMan MGB probes (Applied Biosystems), standardized to VIC labeled *TBP* and *HPRT* TaqMan probes.

### Droplet digital PCR workflow and data analysis

The ddPCR was performed using manufacturer's protocol (Bio-Rad Laboratories), 25 ul of ddPCR mix was prepared using 12.5 ul of Bio-Rad 2× Supermix, 1.25 ul of each of 20× primer/probe mix (Applied Biosystems) along with 60 ng of cDNA, 20 ul of reaction mix was then emulsified with 70 ul of oil using a QX-100 droplet generator according to the manufacturer's instructions (Bio-Rad, Hercules, CA), following PCR amplification droplets were quantified by QX100 droplet reader (Bio-Rad, Hercules, CA). QuantaSoft software version 1.5.38.111 (Bio-Rad, Hercules, CA) was used to quantify the copies/µl of each queried target per well. All samples were run in duplicate for all 10 assays. The gene expression levels measured by ddPCR correlated well with the result of expression array data (Figure S1). The two sets of data had the Pearson's correlation coefficient of r = 0.95, p = 2.6E-5.

### Biological pathways affected in PKS

Using DAVID and GO terms, we quarried for particular classes of genes or biological pathway significantly affected in PKS. The up-regulated genes identified using DAVID included Homeobox, antennepedia type, sequence specific DNA binding, transcription factor activity and genes involved in various developmental processes (Table S3 and S4). Ten out of 180 up-regulated genes were HOX/Homeobox genes, the majority of which were antennapedia- like homeobox- containing *HOXB* (*B2, B3, B5, B6, B7*) and *MEOX2* genes along with the engrailed homeobox family gene *EN1* and the zinc finger homeobox 1b *ZEB2*, mutations in which are associated with Mowat-Wilson syndrome [18].

The genes that are down-regulated by at least 1.5 fold in PKS includes genes involved in anatomical structural morphology and development, various developmental processes and system development and genes regulating signal transduction, cell morphology and cell communication (Table S3 and S4). Several homeobox genes were found to be downregulated. The downregulated homebox genes were mostly from the *HOXA* cluster (*A1, A5, A11, A13*). Homeobox genes *LHX9* and *MEIS2* were also down regulated in the patients as compared to the controls.

**Figure 4:**
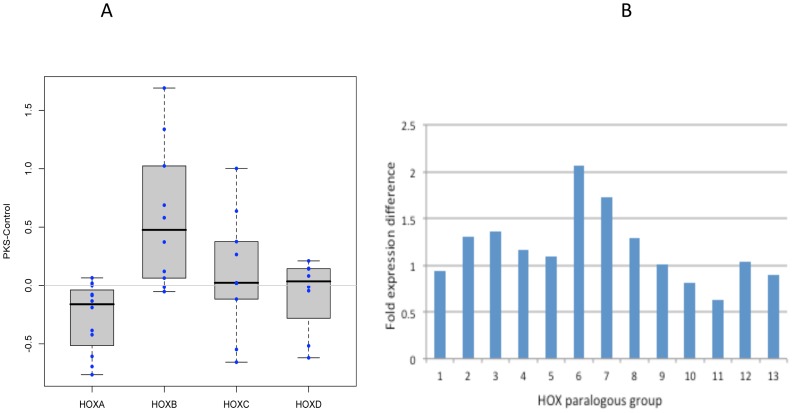
BOX plot of HOX gene clusters. HOX A is down- and HOX B Cluster is up-regulated in PKS patients.

In humans *HOX* genes are found in 4 clusters (A, B, C and D). In probands with PKS, genes of cluster B were up regulated and cluster A was down regulated compared to the controls. Figure 4A is a Box plot of all 4 *HOX* gene clusters. Cluster C and D demonstrated no obvious change in expression level. Mammalian *HOX* gene clusters consist of 13 sets of paralogous group (group 1 to 13), and *HOX* genes belonging to the same paralogous group act synergistically. When we analyze the expression level based on paralogous groups, the central class HOX paralogous group especially paralogous group 6 and 7 were upregulated and abdominal B class HOX paralogous group, especially group 10 and 11 were downregulated throughout HOX cluster A to D (Figure 4B).

Genes were also classified according to their annotated role in biological processes, molecular function and cellular components from GO. Categories such as “development”, “metabolic processes”, “nucleic acid binding”, “gene expression”, “transcription”, “cellular processes and regulation of cell growth”, “cell communication and signal transduction” and “catalytic activities” all demonstrated a higher score.

Among the genes down-regulated the highest proportion corresponded to cell communication, signal transduction and cellular processes along with genes involved in development.

### Functional analysis using IPA

**Figure 5:**
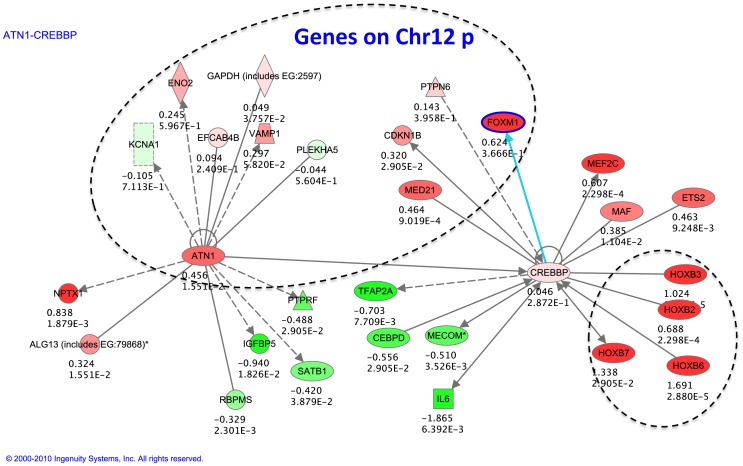
Ingenuity pathway analysis of dysregulated genes in PKS. Green circles represent the genes down-regulated in PKS probands, and red circles represent the genes up-regulated in PKS probands.

The identified genes were analyzed using IPA to investigate functional networks and gene ontology. With the help of ingenuity we were able to establish a molecular link between 12p and the *HOX* genes (Figure 5). *ATN1*, a gene located on 12p13.31, is a transcriptional regulator and is the cause of the dominant neurological disorder dentatorubral pallidoluysian atrophy (DRPLA) when mutated [19], [20]. *ATN1* is up regulated in PKS. It interacts with *CREBBP*, a master transcriptional regulator (and the cause of the developmental disorder Rubinstein-Taybi syndrome, when mutated) [21]. *CREBBP* itself does not demonstrate a significant expression change in PKS, however it interacts with the *HOXB* genes. The dysregulation of *ATN1* may be affecting the dysregulation of the *HOX* genes through the actions of *CREBBP* although further work will be needed to confirm this.

## Discussion

PKS is a multisystem developmental disorder with a highly conserved phenotype. In this study, using the skin fibroblast cell lines derived from 17 probands with PKS, we demonstrate that PKS probands have a unique gene expression signature, which is characterized by misexpression of many developmentally critical genes including multiple homeobox genes. Since many genetic developmental disorders are caused by the perturbation of proper regulation of gene expression during embryogenesis, our observation of a highly conserved pattern of abnormal gene expression of many developmentally critical genes fits very well with the pleiotropic phenotype seen in PKS.

We have previously defined a PKS critical region, whose dosage gain leads to the PKS phenotype [7]. Interestingly, the same region of chromosome 12p, a small 2.80 Mb region on 12p13.31 with 26 genes was identified to contain the most significantly dysregulated gene expression in the PKS probands according to DAVID's analysis, supporting the key role this region likely plays in the pathogenesis of PKS. Out of approximately 100 genes localizing to 12p13.31, *CLEC2B*, *LOC374443* and *NCAPD2* were over-expressed by at least 1.5 fold in the PKS probands. Among these three genes, *NCAPD2* is of particular interest. *NCAPD2* encodes the condensin I complex subunit D2, and plays a well-documented role in regulating the gene expression [22]. Therefore, overexpression of *NCAPD2* may play a central role in the global gene expression abnormality seen in PKS probands as a primary event due to the presence of the isochromosome 12p. In addition to these three genes, chromosomal region 12p13.31 contains additional interesting candidate genes, an extra copy of which could lead to the core phenotype of PKS, although the fold difference of the expression levels were not above 1.5. *ING4* and *CHD4* represent attractive candidate genes responsible for the pleiotropic phenotype seen in PKS, because of the known roles played by these genes in transcriptional regulation. *ING4* encodes a tumor suppressor protein, and plays a role in DNA repair, chromatin remodeling, and regulation of cell growth [23], [24]. The expression level of *ING4* was 1.26 fold higher in PKS probands. *CHD4* is in the same gene family as *CHD7* whose haploinsufficiency leads to CHARGE syndrome, which is a multisystem developmental disorder [25]. *CHD4* is the main component of the nucleosome remodeling and deacetylase complex [26]. *ATN1*, which was highlighted by IPA, also resides at 12p13.31, and mutations of *ATN1* leads to the neurodegenerative disorder DRPLA as discussed above.

The evolutionarily highly conserved *HOX* genes are a subgroup of the homeobox genes. The *HOX* genes are a set of transcription factor encoding genes that pattern the anterior-posterior body axis of animals. The 39 human *HOX* genes are located in four clusters (A–D) on different chromosomes and consists of 9 to 11 genes in each cluster, arranged in tandem that play important roles in embryonic pattering and cell differentiation [27]. The *HOX B* cluster is up- and *HOXA* cluster is down-regulated in the probands, while Cluster C and D had no obvious change in expression level. Such cluster specific changes may implicate the possibility of chromatin modification affecting the entire *HOX* gene cluster in the genome.

When *HOX* gene expression level was classified by paralogous group, interestingly, the central class HOX paralogous group especially paralogous group 6 and 7 were upregulated and abdominal B class HOX paralogous group, especially group 10 and 11, were downregulated throughout HOX cluster A to D. Since the central class of the *HOX* gene groups, and abdominal B class HOX groups, play roles in defining the body segmentation pattern of the thorax and limb, such a dysregulated expression may be associated with the skeletal phenotype of PKS such as vertebral bone/rib anomalies and brachydactyly [28].

Since the most dysregulated genes in PKS were enriched for homebox genes and genes located on 12p, a putative link between the two using IPA was identified. The *ATN1* gene located on 12p interacts with *CREBBP*, a master transcriptional regulator, mutations in which are known to cause Rubinstein Taybi syndrome, no obvious change was noticed in the expression level of *CREBBP* in the probands' tissue studied (skin fibroblast). *CREBBP* also interacts with *HOXB* genes that are upregulated in the probands. It is possible that dysregulation of the *ATN1* gene is affecting the *HOXB* genes modifying the activity of the CREBBP protein without affecting its mRNA level. Further studies need to be done to confirm this hypothesis.

*MED21* was predicted to affect the *HOXB* genes via modulation of the *CREBBP* gene by IPA. *MED21* encodes a member of the mediator complex subunit 21 family, and is involved in transcriptional regulation of RNA polymerase II transcribed genes [29]. The expression level of *MED21* was elevated in PKS probands. Although *MED21* localizes to 12p11.23, which is far from the newly defined PKS critical region, the involvement of *MED21* in the pathogenesis of PKS requires further evaluation, given its critical role in transcriptional regulation.

The downregulated and upregulated genes identified in this expression study highlight many interesting genes whose misexpression may be associated with the PKS phenotype. The most significantly down regulated gene was *ZFPM2* located on chromosome 8q23. Haploinsufficiency of *ZFPM2* is associated with congenital diaphragmatic hernia (CDH) as well as congenital heart defects (CHD), both consistent features of PKS [30], [31]. *GATA6*, located on chromosome 18q11, was also demonstrated to be downregulated in PKS fibroblasts. Mutations of *GATA6* are associated with various types of CHD, hence, *GATA6* may also be associated with the pathogenesis of CHD in PKS [32]. Another gene whose misexpression may be directly related to the phenotype is *SOX9*, because mutations of *SOX9* cause campomelic dysplasia characterized by limb shortening, micrognathia and cleft palate/Robin sequence and sex reversal, and there is phenotypic overlap between campomelic dysplasia and PKS [33], [34]. Therefore, it is tempting to speculate the role of *SOX9* downregulation in the pathogenesis of cleft palate and limb shortening seen in PKS. *IGFBP2* was significantly upregulated in PKS fibroblasts. We hypothesize that overexpression of *IGFBP2* may be associated with the postnatal growth deceleration phenotype seen in PKS. IGFBP2 sequesters free IGF proteins, and functions to mitigate the IGF signaling pathway [35]. Mouse models of *IGFBP2* overexpression demonstrate a postnatal growth retardation phenotype, which is similar to that seen in PKS probands [36].

DAVID annotation was helpful in deciphering which signaling pathways and biological functions are significantly affected in PKS. The terms associated with downregulated and upregulated genes suggests that the pathogenesis of PKS is mainly due to the global abnormalities of transcription and early embryonic development. The term “melanogenesis” was enriched in downregulated genes. This finding is interesting given that PKS is often associated with hypo and hyperpigmented patches (Figure 1). Extra copies of 12p likely cause dysregulated expression of melanogenesis related genes, leading to the pigmentation abnormalities.

**Figure 6:**
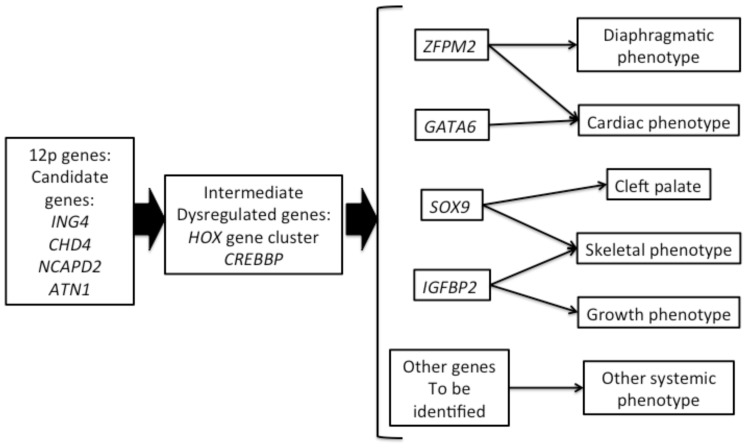
Schematic illustration of disease mechanism of PKS.

In summary, we demonstrate that PKS probands have a unique gene expression profile, which is likely correlated with the highly specific clinical phenotype. While the expression studies described in the experiment were performed on skin fibroblasts and may not be representative of events occurring in early embryonic tissues, several of the identified misexpressed genes provide insight into individual genes and pathways that would appear to be plausible candidates contributing to several of the PKS clinical features (Figure 6). From previous studies, we expect that a single or a few critical genes on 12p, that when present in extra copy numbers, will have a large scale effect on mammalian cell growth and differentiation likely through a large number (cascade) of intermediary genes. Through these studies we are hoping to both identify critical genes on 12p as well as downstream targets in non-12p chromosomal regions.

## Supporting Information

Figure S1 **Scatterplot showing the correlation between 2 technologies (Microarray vs. ddPCR).**
(TIF)

Table S1 **Genes identified as being up-regulated in patients with PKS.**
(XLS)

Table S2 **List of down-regulated genes in patients with PKS.**
(XLS)

Table S3 **DAVID up-regulated pathways.**
(XLS)

Table S4 **DAVID down-regulated pathways.**
(XLS)

Table S5 **List of all Principal Component values.**
(XLSX)

Table S6 **PKS-control difference of 10 differentially expressed genes selected by Microarray data analysis confirmed by ddPCR.**
(XLSX)

## References

[1] Pallister PD, Herrmann J, Meisner LF, Inhorn SI, Opitz JM (1976) Letter: Trisomy-20 syndrome in man. Lancet 1: 431. 10.1016/s0140-6736(76)90276-2 55704

[2] Pallister PD, Meisner LF, Elejalde BR, Francke U, Herrmann J, et al (1977) The pallister mosaic syndrome. Birth Defects Orig Artic Ser 13: 103–110. 890087

[3] Teschler-Nicola M, Killian W (1981) Case report 72: Mental retardation, unusual facial appearance, abnormal hair. Synd Ident 7: 6–7.

[4] Cormier-Daire V, Le Merrer M, Gigarel N, Morichon N, Prieur M, et al (1997) Prezygotic origin of the isochromosome 12p in Pallister-Killian syndrome. Am J Med Genet 69: 166–168. 9056554 10.1002/(sici)1096-8628(19970317)69:2<166::aid-ajmg9>3.0.co;2-n

[5] Peltomaki P, Knuutila S, Ritvanen A, Kaitila I, de la Chapelle A (1987) Pallister-Killian syndrome: cytogenetic and molecular studies. Clin Genet 31: 399–405. 2887316 10.1111/j.1399-0004.1987.tb02832.x

[6] Dufke A, Walczak C, Liehr T, Starke H, Trifonov V, et al (2001) Partial tetrasomy 12pter-12p12.3 in a girl with Pallister-Killian syndrome: extraordinary finding of an analphoid, inverted duplicated marker. Eur J Hum Genet 9: 572–576. 11528501 10.1038/sj.ejhg.5200673

[7] Izumi K, Conlin LK, Berrodin D, Fincher C, Wilkens A, et al (2012) Duplication 12p and Pallister-Killian syndrome: a case report and review of the literature toward defining a Pallister-Killian syndrome minimal critical region. Am J Med Genet A 158A: 3033–3045. 23169682 10.1002/ajmg.a.35500

[8] Liehr T, Claussen U, Starke H (2004) Small supernumerary marker chromosomes (sSMC) in humans. Cytogenet Genome Res 107: 55–67. 15305057 10.1159/000079572

[9] Wilkens A, Liu H, Park K, Campbell LB, Jackson M, et al (2012) Novel clinical manifestations in Pallister-Killian syndrome: comprehensive evaluation of 59 affected individuals and review of previously reported cases. Am J Med Genet A 158A: 3002–3017. 23169767 10.1002/ajmg.a.35722

[10] Leube B, Majewski F, Gebauer J, Royer-Pokora B (2003) Clinical, cytogenetic, and molecular observations in a patient with Pallister-Killian-syndrome with an unusual karyotype. Am J Med Genet A 123A: 296–300. 14608653 10.1002/ajmg.a.20339

[11] Schubert R, Viersbach R, Eggermann T, Hansmann M, Schwanitz G (1997) Report of two new cases of Pallister-Killian syndrome confirmed by FISH: tissue-specific mosaicism and loss of i(12p) by in vitro selection. Am J Med Genet 72: 106–110. 9295085 10.1002/(sici)1096-8628(19971003)72:1<106::aid-ajmg21>3.0.co;2-u

[12] Conlin LK, Kaur M, Izumi K, Campbell L, Wilkens A, et al (2012) Utility of SNP arrays in detecting, quantifying, and determining meiotic origin of tetrasomy 12p in blood from individuals with Pallister-Killian syndrome. Am J Med Genet A 158A: 3046–3053. 23169773 10.1002/ajmg.a.35726

[13] Schinzel A (1991) Tetrasomy 12p (Pallister-Killian syndrome). J Med Genet 28: 122–125. 2002482 10.1136/jmg.28.2.122 PMC1016781

[14] Horn D, Majewski F, Hildebrandt B, Korner H (1995) Pallister-Killian syndrome: normal karyotype in prenatal chorionic villi, in postnatal lymphocytes, and in slowly growing epidermal cells, but mosaic tetrasomy 12p in skin fibroblasts. J Med Genet 32: 68–71. 7897632 10.1136/jmg.32.1.68 PMC1050184

[15] DeScipio C, Kaur M, Yaeger D, Innis JW, Spinner NB, et al (2005) Chromosome rearrangements in cornelia de Lange syndrome (CdLS): report of a der(3)t(3;12)(p25.3;p13.3) in two half sibs with features of CdLS and review of reported CdLS cases with chromosome rearrangements. Am J Med Genet A 137A: 276–282. 16075459 10.1002/ajmg.a.30857 PMC4896149

[16] Conlin LK, Thiel BD, Bonnemann CG, Medne L, Ernst LM, et al (2010) Mechanisms of mosaicism, chimerism and uniparental disomy identified by single nucleotide polymorphism array analysis. Hum Mol Genet 19: 1263–1275. 20053666 10.1093/hmg/ddq003 PMC3146011

[17] Bjork KE, Kafadar K (2007) Systematic order-dependent effect in expression values, variance, detection calls and differential expression in Affymetrix GeneChips. Bioinformatics 23: 2873–2880. 17897972 10.1093/bioinformatics/btm450

[18] Garavelli L, Mainardi PC (2007) Mowat-Wilson syndrome. Orphanet J Rare Dis 2: 42. 17958891 10.1186/1750-1172-2-42 PMC2174447

[19] Koide R, Ikeuchi T, Onodera O, Tanaka H, Igarashi S, et al (1994) Unstable expansion of CAG repeat in hereditary dentatorubral-pallidoluysian atrophy (DRPLA). Nat Genet 6: 9–13. 8136840 10.1038/ng0194-9

[20] Nagafuchi S, Yanagisawa H, Sato K, Shirayama T, Ohsaki E, et al (1994) Dentatorubral and pallidoluysian atrophy expansion of an unstable CAG trinucleotide on chromosome 12p. Nat Genet 6: 14–18. 8136826 10.1038/ng0194-14

[21] Petrij F, Giles RH, Dauwerse HG, Saris JJ, Hennekam RC, et al (1995) Rubinstein-Taybi syndrome caused by mutations in the transcriptional co-activator CBP. Nature 376: 348–351. 7630403 10.1038/376348a0

[22] Aragon L, Martinez-Perez E, Merkenschlager M (2013) Condensin, cohesin and the control of chromatin states. Curr Opin Genet Dev 23: 204–211. 23312842 10.1016/j.gde.2012.11.004

[23] Nozell S, Laver T, Moseley D, Nowoslawski L, De Vos M, et al (2008) The ING4 tumor suppressor attenuates NF-kappaB activity at the promoters of target genes. Mol Cell Biol 28: 6632–6645. 18779315 10.1128/MCB.00697-08 PMC2573235

[24] Hung T, Binda O, Champagne KS, Kuo AJ, Johnson K, et al (2009) ING4 mediates crosstalk between histone H3 K4 trimethylation and H3 acetylation to attenuate cellular transformation. Mol Cell 33: 248–256. 19187765 10.1016/j.molcel.2008.12.016 PMC2650391

[25] Vissers LE, van Ravenswaaij CM, Admiraal R, Hurst JA, de Vries BB, et al (2004) Mutations in a new member of the chromodomain gene family cause CHARGE syndrome. Nat Genet 36: 955–957. 15300250 10.1038/ng1407

[26] Tong JK, Hassig CA, Schnitzler GR, Kingston RE, Schreiber SL (1998) Chromatin deacetylation by an ATP-dependent nucleosome remodelling complex. Nature 395: 917–921. 9804427 10.1038/27699

[27] Pearson JC, Lemons D, McGinnis W (2005) Modulating Hox gene functions during animal body patterning. Nat Rev Genet 6: 893–904. 16341070 10.1038/nrg1726

[28] Jamuar S, Lai A, Unger S, Nishimura G (2012) Clinical and radiological findings in Pallister-Killian syndrome. Eur J Med Genet 55: 167–172. 22387057 10.1016/j.ejmg.2012.01.019

[29] Kremer SB, Kim S, Jeon JO, Moustafa YW, Chen A, et al (2012) Role of Mediator in regulating Pol II elongation and nucleosome displacement in Saccharomyces cerevisiae. Genetics 191: 95–106. 22377631 10.1534/genetics.111.135806 PMC3338273

[30] Ackerman KG, Herron BJ, Vargas SO, Huang H, Tevosian SG, et al (2005) Fog2 is required for normal diaphragm and lung development in mice and humans. PLoS Genet 1: 58–65. 16103912 10.1371/journal.pgen.0010010 PMC1183529

[31] Pizzuti A, Sarkozy A, Newton AL, Conti E, Flex E, et al (2003) Mutations of ZFPM2/FOG2 gene in sporadic cases of tetralogy of Fallot. Hum Mutat 22: 372–377. 14517948 10.1002/humu.10261

[32] Kodo K, Nishizawa T, Furutani M, Arai S, Yamamura E, et al (2009) GATA6 mutations cause human cardiac outflow tract defects by disrupting semaphorin-plexin signaling. Proc Natl Acad Sci U S A 106: 13933–13938. 19666519 10.1073/pnas.0904744106 PMC2728998

[33] Foster JW, Dominguez-Steglich MA, Guioli S, Kwok C, Weller PA, et al (1994) Campomelic dysplasia and autosomal sex reversal caused by mutations in an SRY-related gene. Nature 372: 525–530. 7990924 10.1038/372525a0

[34] Wagner T, Wirth J, Meyer J, Zabel B, Held M, et al (1994) Autosomal sex reversal and campomelic dysplasia are caused by mutations in and around the SRY-related gene SOX9. Cell 79: 1111–1120. 8001137 10.1016/0092-8674(94)90041-8

[35] Firth SM, Baxter RC (2002) Cellular actions of the insulin-like growth factor binding proteins. Endocr Rev 23: 824–854. 12466191 10.1210/er.2001-0033

[36] Hoeflich A, Wu M, Mohan S, Foll J, Wanke R, et al (1999) Overexpression of insulin-like growth factor-binding protein-2 in transgenic mice reduces postnatal body weight gain. Endocrinology 140: 5488–5496. 10579311 10.1210/endo.140.12.7169

