# Supplementary figures and images for: Genome-Wide Expression Analysis in Fibroblast Cell Lines from Probands with Pallister Killian Syndrome
Source: PLoS One. 2014 Oct 16;9(10):e108853. doi: 10.1371/journal.pone.0108853 (PMC4199614; doi:10.1371/journal.pone.0108853)

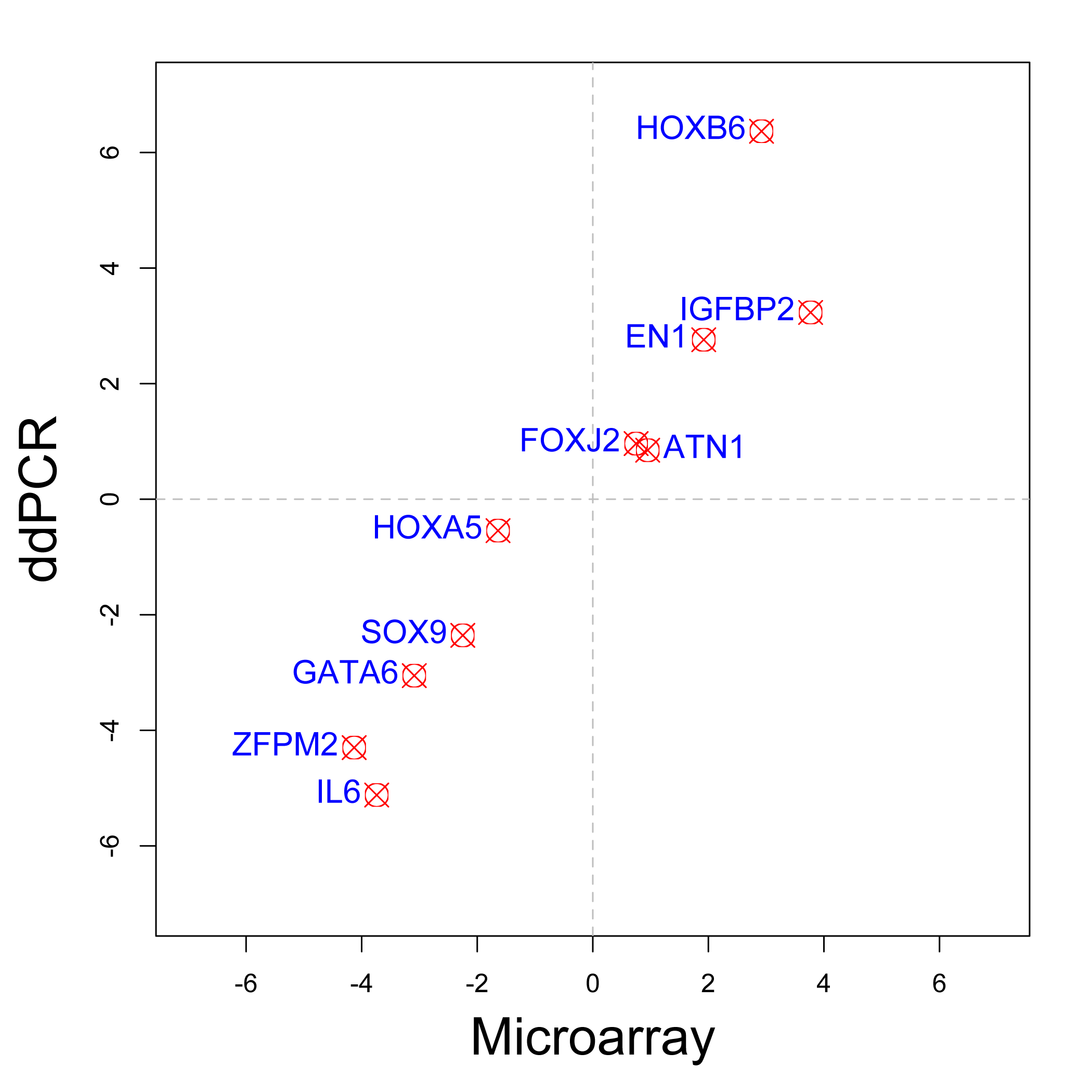

Supplement: Figure S1 — Scatterplot showing the correlation between 2 technologies (Microarray vs. ddPCR). (TIF) [file pone.0108853.s001.tif]
